# Laughter and Short-Term Blood Pressure Reactivity in Spousal Support Interactions

**DOI:** 10.1093/geroni/igaa057.2236

**Published:** 2020-12-16

**Authors:** Joan Monin, Jennifer Tomlinson, Brooke Feeney

**Affiliations:** 1 Yale University, New Haven, Connecticut, United States; 2 Colgate University, Hamilton, New York, United States; 3 Carnegie Mellon University, Pittsburgh, Pennsylvania, United States

## Abstract

Individual effects of laughter in reducing stress are well-documented. However, no research has examined dyadic associations between laughter and blood pressure in spousal support interactions. This study examined the hypotheses that individual and shared laughter would be associated with lower blood pressure and distress during a support interaction for both the “support-seeker” and the “support-provider”. Two hundred and seventy-one older adult couples were video-recorded and their blood pressure was monitored during a baseline, a discussion about the support-seeker’s greatest fear related to aging, and while playing a game in the laboratory. Both spouses reported their distress after the support interaction. Laughter was coded by trained observers. According to the Actor Partner Interdependence Models, the more the support-seeker laughed, the lower the support-provider’s systolic blood pressure was during the support interaction (partner effect). Also, laughter was associated with less distress for both spouses during the support interaction (actor effects). Part of a symposium sponsored by Dyadic Research on Health and Illness Across the Adult Lifespan Interest Group.

